# Safety, Efficacy, and Predictive Factors of Conventional Epithelium-Off Corneal Crosslinking in the Treatment of Progressive Keratoconus

**DOI:** 10.1155/2020/7487186

**Published:** 2020-05-31

**Authors:** Rebecca Farhat, Mohamad Khaled Ghannam, Georges Azar, Joseph Nehme, Marwan Sahyoun, Najib Georges Hanna, Mariana Abi Karam, Charbel El Haber, Alexandre Schakal, Alexandre Jalkh, Ameed Samaha

**Affiliations:** ^1^Eye and Ear Hospital International, Naccach, Lebanon; ^2^Holy-Spirit University, Kaslik, Lebanon; ^3^Saint-Joseph University, Beirut, Lebanon

## Abstract

**Purpose:**

To report predictive factors of outcome of conventional epithelium-off corneal crosslinking (CXL) in the treatment of progressive keratoconus.

**Methods:**

This is a monocentric observational retrospective study conducted at Eye and Ear International Hospital, Lebanon. All patients with progressive keratoconus who underwent CXL between January 2008 and January 2016, with minimal 3-years follow-up were included. Primary treatment outcomes were maximum keratometry (K max), best-corrected distance visual acuity (CDVA), and failure. Failure was defined as an increase of 1.00 diopters (D) or more in K max and/or an increase of 0.1 logMAR or more in CDVA and conversion to corneal transplantation. Statistical analysis was done to identify predictors of treatment success. Univariate and multivariate analyses were performed to determine the correlations between baseline parameters and outcomes, and an equation for predicting K max and CDVA was created.

**Results:**

156 eyes of 102 patients were enrolled. The mean age was 23.85 ± 6.52 years. Failure occurred in 31 eyes (19.87%). Gender and thinnest pachymetry did not have any impact on postoperative outcomes. Concerning the CDVA outcome, multivariate analysis showed that a better preoperative CDVA was associated with higher improvement in CDVA, and higher baseline K max and higher posterior mean K were associated with a worse outcome CDVA. Regarding postoperative K max, a higher baseline K max, a worse baseline CDVA, and a younger age were associated with less flattening postoperatively.

**Conclusion:**

CXL is a safe and effective method in treating progressive keratoconus. However, the clinical benefits can differ among patients, and in our series, a nonnegligible number of cases show a continued progression of their ectasia. Further studies to identify predictors of postoperative progression prior to the procedure could help sort out good responders to treatment.

## 1. Introduction

Corneal ectasia is a degenerative noninflammatory eye condition characterized by a loss of corneal stability, which leads to progressive thinning and bulging of the cornea. Primary forms include mainly keratoconus (KC), keratoglobus, and pellucid marginal degeneration, while acquired forms are mostly a complication of refractive surgery [1].

The estimated incidence of KC varies between 1/500 and 1/2,000 individuals in the general population with higher numbers among Asians and the Caucasian population. The classical presentation of KC is to have onset during the late teens or early twenties with a variable course of progression of corneal bulging until the 4^th^ decade, having a significant negative impact on the quality of life and life planning [2].

Treatment options for keratoconus consist of wearing spectacles and soft or rigid contact lenses (CL) [3]. For those who cannot tolerate CL wear or do not achieve a good vision with spectacles or CL, the implantation of an intracorneal ring segment (ICRS) may be advised. Furthermore, in some advanced cases with severe corneal scarring or thinning, corneal transplantation like deep anterior lamellar keratoplasty (DALK) or penetrating keratoplasty (PK) may be the unique option to restore a good vision [4]. However, none of those treatments aim to counter the progression of the disease [5].

Corneal collagen crosslinking (CXL) was introduced in the late decades of the 20^th^ century and received the Food Drug Administration (FDA) approval in April 2016 for treating progressive KC and secondary corneal ectasia based on three 12-months clinical trials [6]. However, continued disease progression and an additional decline in visual acuity have been noted postoperatively [7].

In this paper, we aim to share our experience with CXL in treating keratoconus, safety, efficacy, and predictors for treatment outcomes.

## 2. Methods

### 2.1. Data Set and Study Design

In this retrospective study, the medical charts of consecutive patients who underwent conventional epithelium-off corneal CXL for progressive KC at the Eye and Ear Hospital International, Lebanon, from January 2008 to January 2016 were reviewed.

CXL was indicated in all patient based on the progression rate of their ectasia: an increase in K max at the apex of 1.00 D or more in 1 year, a change in astigmatism ≥3 D in 6 months or the need for new CL fitting more than once in two years [8]. The inclusion criteria were thinnest corneal pachymetry ≥400 *μ*m, a clear cornea on slit lamp, and an adequate follow-up of 3 years. Patients with a history of previous corneal surgery, severe corneal scarring or infection, diseases of the lens or the retina, history of poor epithelial wound healing, pregnant and lactating women, and those who had a refractive surgery or intracorneal ring segment implantation at any time were excluded.

A study license was obtained from the local ethics committee and the study was performed in keeping with the guidelines of the Declaration of Helsinki.

The following primary outcomes were recorded preoperatively and at the last follow-up visit: (1) LogMAR corrected distance visual acuity (logMar CDVA), (2) topographic indices (anterior flat-K and steep-K of the central 3 mm of the cornea, K max at the apex, posterior mean K and thinnest pachymetry (TP)) with Allegro Oculyzer (WaveLight, GmbH, Erlangen, Germany), (3) a full ocular examination including slit lamp biomicroscopy to detect any ocular complication of treatment. CL wearers were instructed to stop their use two weeks before their visit to avoid false K values and warpage. Treatment failure was defined by an increase in K max of 1.00 D or more and/or increase in CDVA of at least 0.1 logMar [9] at the last visit or conversion to transplantation. The following complications were looked for: corneal burn, persisting corneal haze, infectious keratitis, corneal edema due to endothelial failure, corneal melting and perforation, corneal scarring, herpes reactivation, and limbal stem cell deficiency.

### 2.2. Surgical Procedure

All interventions were performed by the same surgeon, under controlled and sterile conditions, and according to the conventional standardized epithelium-off technique, Dresden protocol. After applying topical anesthesia, the central corneal epithelium of 8.0 to 9.0 mm was removed with a blunt spatula and a standard isotonic riboflavin solution 0.1% (LightMed Collagex; Australia) was instilled every 2 minutes over 30-minute period. All patients were examined at the slit lamp to ensure the penetration of riboflavin into the anterior chamber, and their central corneal thickness was measured by ultrasound before starting Ultraviolet-A (UVA) irradiation. Light is emitted at a wavelength of 365 ± 10 nm and an irradiance of 3 mW/cm^2^ or 5.4 J/cm^2^ from a solid-state device (UV-X 1000; IROC Innocross, Zug, Switzerland). Minimum of 400 *μ*m central corneal thickness was considered at the time of UVA irradiation. In the event of central corneal thickness below 400 *μ*m, we used hypoosmotic riboflavin to swell the cornea before starting UVA irradiation. Riboflavin acts as a photosensitizer by increasing UVA absorption by the cornea. Irradiation with UVA of 370 nm and 5.4 J/cm^2^ is applied for another 30 minutes while instilling 0.1% Riboflavin every 3 minutes. At the end of the procedure, the eye is washed with sterile saline solution, and a bandage lens (BL) was applied and removed 3 days following treatment or upon complete closure of the epithelial abrasion. Patients were treated postoperatively with preservative free Levofloxacin eye drops and dexamethasone eye drops, both 3 times per day. Lubricating eye drops were also prescribed. Levofloxacin eye drops were stopped 2 days after BL removal, and dexamethasone eye drops were administered for a total 2 weeks tapering regimen. Preservative free artificial tears were prescribed for at least 3 months and PRN afterwards.

### 2.3. Statistical Analysis

Data were collected and regrouped in a database program on a personal computer. Statistical analysis was performed using commercially available software (SPSS version 20.0, SPSS Inc., Chicago, Illinois). Continuous variables were noted by means and their corresponding standard deviation (SD). The paired Student's *t*-test was used to study the variation in K max, Steep-K, Flat-K, and logMAR CDVA between baseline and the last visit. Assuming a normal distribution of the data, ANOVA F-test was used to compare the failure and success. Statistical significance was set at *p* values of <0.05 at a 95% confidence interval.

Univariable analysis was performed in an attempt to determine predictor factors of the primary outcomes: change in K max and CDVA. Factors with *p* ≤ 0.20 from the univariable analysis were all used in a multivariate linear regression analysis to determine independent predictive factors. Generalized estimating equations were used to perform the analysis. A correction was done for patients who received bilateral treatment.

The prediction model was validated. The predicted and observed variations in K max and in logMAR CDVA values were compared according to linear regression and presented in a calibration plot.

## 3. Results

### 3.1. Demographic Characteristics

Our study included 156 eyes from 102 patients who underwent standard epithelium-off corneal CXL according to Dresden protocol. The study involved a greater proportion of men 106 (68%) than women 50 (32%). The mean patients' age at the time of the procedure was 23.85 ± 6.52 years old (range, 9–41), and follow-up duration was 3 years (Table 1).

### 3.2. Overall Outcomes at the Last Visit

**Figure 1:**
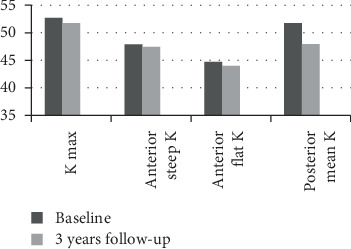
Mean keratometric values before and at the last follow-up after corneal crosslinking.

Table 2 and Figure 1 show the variations in different mean parameters between baseline and the last visit after crosslinking and the correspondent *p* value for the whole study participants.

By the end of 3 years follow-up there was a significant flattening in posterior and anterior corneal curvature (*p* < 0.01^*∗*^). The TP was significantly decreased between baseline and the last visit (*p* < 0.01^*∗*^).

The mean preoperative CDVA significantly improved by 0.022 logMAR (*p*=0.033) at the end of the follow-up. In all eyes, the postoperative CDVA was recorded using spectacles or hard contact lenses in concordance with the visual acuity exam before CXL.

### 3.3. Comparison between the Subgroups according to Failure

Failure was considered if patients presented an increase in K max value by 1 D or more and/or an increase of more than 0.1 in logMAR CDVA during the follow-up or conversion to corneal transplantation. In our series, failure occurred in 31 eyes (19.87%). Five eyes (3.20%) presented with an increase in logMAR CDVA of more than 0.1, 14 eyes (8.97%) presented with an increase in K max by more than 1 D, 12 eyes (7.69%) met these two criteria, and 1 eye (0.65%) underwent corneal transplantation postoperatively.

Further statistical analyses were done to identify predictors of the effectiveness of corneal crosslinking. Flatter anterior and flatter posterior corneal curvatures (anterior flat-K and steep-K of the central 3 mm of the cornea, K max at the apex and posterior mean K) were found to be independent factors predicting a successful treatment. Higher baseline pachymetry and older age were also identified as significant predictors of treatment success. Baseline visual acuity and gender did not have any significant impact on the treatment outcome (Table 3).

### 3.4. Univariate Analysis

Table 4 summarizes the univariate correlation between the presumed predictor's baseline and post-CXL CDVA and K max (dependent predictive variable). Gender and TP did not have any impact on postoperative outcomes. Predictors of the CDVA changes included CDVA (*p* < 0.01), K max (*p* < 0.01), and posterior mean (K). On the other hand, K max value variation was related significantly to baseline CDVA (*p* < 0.01), baseline K max (*p* < 0.01), and age (*p* < 0.01). The significant values, B coefficient, and a 95% confidence interval are presented in Table 4. The significant univariate associations were entered in the multivariate analysis.

### 3.5. Multivariate Analysis

Concerning the CDVA outcome, baseline CDVA (logMAR) (*p* < 0.01), baseline K max (*p* < 0.01), and baseline posterior mean K were the only independent predictors. This means that a better preoperative CDVA would be associated with higher improvement in CDVA and a higher baseline K max and higher posterior mean K were associated with a worse outcome CDVA. Regarding the postoperative corneal flattening, baseline K max, baseline CDVA, and age were identified as independent predictors. This means a higher baseline K max, a worse baseline CDVA and a younger age were associated with less flattening postoperatively (Table 5).

### 3.6. Prediction Equations

To predict the postoperative CDVA (logMAR) and the postoperative K max at the last follow-up, these equations were applied.

(1) $$\begin{array}{c} \text{Postoperative CDVA}=-0.408+0.01x\text{ baseline posterior mean K}-0.03x\text{ age }\left(\text{years}\right)+0.446x\text{ baseline CDVA}\\ \text{Postoperative K max}=15.202+0.821x\text{ baseline K max}-0.111x\text{ age }\left(\text{years}\right)+8.925x\text{ baseline CDVA.} \end{array}$$

### 3.7. Complications

Relevant complications such as infectious keratitis, sterile infiltrate, corneal edema due to endothelial decompensation, corneal melting and perforation, prolonged reepithelialization, and limbal stem cell insufficiency were not observed during the follow-up period. However, one eye (0.64%) developed a stromal corneal scar that affected CDVA postoperatively. The patient who developed stromal scarring had a stage 1 keratoconus based on Amsler-Krumeich classification [10]. His mean central K readings was 44.5 D, his thinnest pachymetry was 466 *μ*m, and his myopia and astigmatism were less than 8.00 D without any baseline corneal scar. In addition, the persistent anterior stromal haze was noted at the last follow-up in 19 eyes (12.18%). The slit lamp examination grading was as follows: (0) clear cornea; (1) focal areas of minimal stromal clouding or reticulation; (2) diffuse mild stromal clouding or reticulation; (3) diffuse stromal clouding or reticulation somewhat obscuring the view of iris details; (4) focal or diffuse areas of dense stromal haze obscuring iris details [11]. According to this scale, 14 eyes in our study had persistent grade 1 haze, 3 eyes had persistent grade 2 haze, and 2 eyes had persistent grade 3 haze. In eyes with persistent grade 2 and 3, corneal haze CDVA was affected by 0.1 or more increase in logMAR. We did not find any significant correlation between baseline characteristics and haze formation, including K max at the apex, posterior and anterior curvature, TP, and age.

## 4. Discussion

Corneal crosslinking is actually the unique conservative treatment of corneal ectatic pathologies aiming to slow or even to stop the evolution of the disease. Many CXL protocols have been developed, but the conventional epithelium-off CXL remains the most effective [12]. Studies on corneal CXL go back more than a decade, and the US Food and Drug Administration (FDA) approved this technique in April 2016, years after European countries. To date, several studies showed long-term stability after corneal crosslinking without serious side effects [13–15]. However, continued disease evolution and a further decline in visual acuity have been reported following treatment [7]. In addition, studies concerning predictors of success for crosslinking are not conclusive yet, which increases the need for more work on this aspect.

Indeed, for Poli et al. 6 years postoperatively, significant improvement in CDVA and long-term stability of topographic indices was observed in a cohort of 36 eyes [16]. Similarly, Caporosi et al. reported significant amelioration of uncorrected distance visual acuity (UDCVA), CDVA, K max, and kmin in a cohort of 44 eyes with a success rate of 100% in terms of stopping the progression of corneal ectasia [17]. Contrarily, Koller et al. conducted a prospective study of 117 eyes and reported a 2.9% loss of 2 or more Snellen lines of CDVA and a 7.6% increase in the K max of more than 1 D over the baseline value [7].

In our nonrandomized retrospective study, treatment with CXL showed a significant decrease in the mean maximum and minimum corneal curvature of the central 3 mm of the cornea anteriorly (steep-K and flat-K), the mean curvature at the apex of the cone (K max), and the posterior mean K. The mean CDVA also showed a significant improvement of 0.022 logMAR (*p*=0.033), which can be attributed to the reduction of corneal curvature and distortion and a better fit of hard contact lenses enabled by the homogenization of the corneal surface. These findings agree with the reported results in a larger case series, which studied 241 keratoconic eyes between 3 and 6 years [8]. In that retrospective study, a significant amelioration was observed in CDVA and maximum K.

Additionally, the mean TP showed a significant reduction than the baseline values (*p* < 0.01^*∗*^). Other studies reported the same results, suggesting that the postoperative thinning may be due to compression of collagen fibrils, changes in corneal hydration and edema, and keratocyte apoptosis [15, 18, 19]. On the contrary, corneal thickness remained unchanged in other series [11]. Accordingly, we can say that corneal pachymetry post-CXL is no longer reliable to assess disease progression.

We also demonstrated that after a follow-up of 3 years, the progression of keratoconus was stopped or improved in 120 eyes (76.92%).

On the other hand, 31 (19.87%) eyes showed continued progression with an increase in K max of 1.0 D over the baseline value and/or an increase of 0.1 or more in logMAR CDVA and 1 patient underwent a penetrating keratoplasty. Thus, the failure rate of the intervention was 19.87% (31 eyes), in accordance with available data in the literature, where treatment failure occurred in 8.1–33.3% of the cases [17]. Many authors question these statistics because they consider that failure definitions in the literature were not consistent [20]. We could correlate the relatively high percentage of failure in our study to our choice of strict success criteria. An increase in K max of 1 D or more at the last visit was seen in 26 eyes. However, only 12 eyes had a functional impact with an increase of logMAR visual acuity and for the remainder, it was only a morphological variation.

Parameters at baseline were also studied in an attempt to identify predictors of postoperative overall progression. In the current study, gender and TP did not have any significant impact on the treatment outcomes regarding K max and CDVA. Concerning the postoperative CDVA, a better baseline CDVA and a lower K max and lower posterior mean K were good predictors for postoperative improvement in CDVA. However, age did not show any influence on the CDVA outcome.

Regarding the postoperative K max, better baseline CDVA, lower K max, and an advanced age seemed to be significant predictors of postoperative flattening.

Regarding K max, our current study validates the findings of Raiskup and Spoerl [5] but is in contrast with other published studies, which elucidated more prominent corneal flattening postoperatively in ectasia cases with higher preoperative K max [21–24] or an insignificant impact of baseline K max [25, 26].

Based on our results, a better baseline CDVA was a significant predictor for improvement regarding outcome K max and outcome CDVA. These results were inconsistent with many previous studies which reported that a worse baseline CDVA was a good predictor of both visual and topographic improvements [26] and with those who reported an insignificant effect of baseline CDVA on postoperative K max after crosslinking [2].

Pachymetry in our series had no significant impact on outcome K and CDVA when each criterion was taken alone. However, patients in whom CXL failed had a thinner cornea (*p* < 0.01^*∗*^) when failure was defined as the presence of one or both criteria. Studies in the literature contradict one another concerning pachymetry; findings of Godefrooij et al. are comparable with ours [21], Badawi et alshowed in their study that thinner corneas were good predictors for postoperative CDVA improvement only without any effect on keratometry [18] while Toprak et al. stated that thinner corneas exhibited more flattening post-CXL [26].

Age is also debated as an independent factor for predicting the outcome of treating keratoconus with CXL. Our results concerning patient age at baseline were consistent with a previous study by Toprak et al. [26], who concluded that older patients had more postoperative corneal flattening compared with the results of the younger patient but, on the other hand, Soeters et al. [22] and Godefrooij et al. [21] reported better postoperative outcomes in younger patients.

We did not note any early complications such as infectious keratitis, persistent epithelial defect, and corneal burn. However, one eye had postoperative vision limiting stromal scarring (0.64%) and 19 (12.18%) had persistent anterior stromal haze responsible for 5 cases (3.20%) of decreased visual acuity, whose CDVA increased by 0.1 or 0.2 logMAR. In fact, corneal haze is a relatively common complication of standard epi-off corneal crosslinking reported by 10–90% of patients [9]. In our group, we did not find any correlation between baseline characteristics and haze formation, although some studies reported an overall increase of haze formation in eyes with very high preoperative K max values as seen in the advanced stage of ectasia [27]. However, CXL associated corneal haze might be a sign of the efficacy of the treatment action or, conversely, an adverse event. Further studies, assessing for example, low contrast acuity or contrast sensitivity, may help clarify the clinical implication of the haze [27].

Our study limitations include mainly the design (retrospective). We did not analyze other outcomes such as endothelial cell count variation due to a lack of data. In addition, some clinical outcomes are subjective such as biomicroscopic exam and grading of corneal haze and were being evaluated by multiple investigators.

## 5. Conclusion

In summary, our results indicate that CXL is effective and safe in stopping the progression of keratoconus in most of the eyes with some improvement in visual acuity due to a reduction in corneal topographic measurements. However, the clinical benefits of CXL can differ among patients, and in our series, a nonnegligible number of cases show a continued progression of their ectasia. A better preoperative CDVA was associated with higher improvement in CDVA, and higher baseline K max and higher posterior mean K were associated with a worse outcome CDVA, while a higher baseline K max, a worse baseline CDVA, and a younger age were associated with less flattening postoperatively. Therefore, further studies aiming to identify predictors of postoperative progression prior to the procedure are crucial to help physicians manage their patient's expectations and lower the exposure to potential adverse events.

## Figures and Tables

**Table 1:** Demographic data of the patients.

Total number	156 eyes
Sex	106 males (68%)
	50 females (32%)
Age (years)	23.85 ± 6.52 years
Follow-up	3 years
Laterality	54 patients (bilateral) (52.94%)
	48 patients (unilateral) (47.05%)

**Table 2:** The changes in mean different topographic parameters between baseline and the last follow-up after crosslinking and their *p* value.

Parameters	Baseline mean ± SD	Last visit mean ± SD	*p* value
CDVA (logMAR)	0.129 ± 0.14	0.107 ± 0.14	0.033^*∗*^
K Max (D)	52.67 ± 5.93	51.72 ± 6.18	<0.01^*∗*^
Anterior steep-K (D)	47.88 ± 4.18	47.42 ± 4.43	<0.01^*∗*^
Anterior flat-K (D)	44.68 ± 2.90	43.99 ± 3.54	<0.01^*∗*^
Posterior mean K (D)	51.75 ± 4.47	47.92 ± 6.33	<0.01^*∗*^
Thinnest pachymetry (*μ*m)	481.03 ± 48.46	461.94 ± 56.25	<0.01^*∗*^

^∗^ *p* significant at the value <0.05.

**Table 3:** Comparison between success and failure subgroups according to baseline characteristics.

	Failure group	Success group	*p* value
Age	22.02 ± 6.23	24.61 ± 6.50	0.023^*∗*^
CDVA (logMAR)	0.14 ± 0.13	0.12 ± 0.14	0.540
K max (D)	54.84 ± 4.66	51.73 ± 6.14	<0.01^*∗*^
Anterior steep-K (D)	49.56 ± 3.76	47.17 ± 4.15	<0.01^*∗*^
Anterior flat-K (D)	45.43 ± 2.65	44.37 ± 2.95	0.038^*∗*^
Posterior mean K (D)	53.37 ± 4.14	51.07 ± 4.44	<0.01^*∗*^
Thinnest pachymetry (*μ*m)	464.31 ± 43.95	493.27 ± 40.11	<0.01^*∗*^
Gender			
Male	32	74	0.981
Female	15	35	

^∗^ *p* significant at the value of 0.05.

**Table 4:** Univariate linear regression of the baseline predictive factors and its impact on the treatment outcome.

Parameters	Univariate analysis				Univariate analysis			
	Dependent variable: CDVA post-CXL				Dependent variable: K max post-CXL			
	B	Significant *p* value	95% confidence interval		B	Significant *p* value	95% confidence interval	
			Lower bound	Upper bound			Lower bound	Upper bound
CDVA	0.0505	<0.01^*∗*^	0.363	0.648	5.938	<0.01^*∗*^	2.383	9.494
K max	0.006	<0.01^*∗*^	0.003	0.009	0.844	<0.01^*∗*^	0.760	0.927
Age	–0.001	0.354	–0.004	0.001	–0.154	<0.01^*∗*^	–0.223	–0.086
Male sex	–0.200	0.305	–0.059	0.018	–0.203	0.677	–1.163	0.757
Posterior mean K	0.100	0.027^*∗*^	0.001	0.019	0.022	0.845	–0.201	0.244
Thinnest pachymetry	0.000	0.605	–0.001	0.000	–0.12	0.114	–0.27	0.003

^*∗*^This parameter is set to zero because it is redundant.

**Table 5:** Multivariate linear regression of the baseline predictive factors and its impact on the treatment outcomes.

Multivariate analysis				
Dependent variable	B	Significant *p* value	95% confidence interval	
			Lower bound	Upper bound
K max post				
Age	–0.154	<0.01^*∗*^	–0.222	–0.86
K max	0.845	<0.01^*∗*^	0.762	0.929
CDVA	5.886	<0.01^*∗*^	2.347	9.424
Posterior mean K	0.005	0.094	–0.001	0.011
CDVA post				
Age	–0.001	0.349	–0.004	0.001
K max	0.006	<0.01^*∗*^	0.003	0.010
CDVA	0.502	<0.01^*∗*^	0.360	0.644
Posterior mean K	0.183	0.13^*∗*^	0.040	0.326

## Data Availability

The excel data used to support the findings of this study are available from the corresponding author upon request.
